# Biometric From Surface Electromyogram (sEMG): Feasibility of User Verification and Identification Based on Gesture Recognition

**DOI:** 10.3389/fbioe.2020.00058

**Published:** 2020-02-14

**Authors:** Jiayuan He, Ning Jiang

**Affiliations:** Department of Systems Design Engineering, Faculty of Engineering, University of Waterloo, Waterloo, ON, Canada

**Keywords:** biometrics, gesture recognition, surface electromyogram, user verification, user identification

## Abstract

Electrical biosignals are favored as biometric traits due to their hidden nature and allowing for liveness detection. This study explored the feasibility of surface electromyogram (sEMG), the electrical manifestation of muscle activities, as a biometric trait. The accurate gesture recognition from sEMG provided a unique advantage over two traditional electrical biosignal traits, electrocardiogram (ECG), and electroencephalogram (EEG), enabling users to customize their own gesture codes. The performance of 16 static wrist and hand gestures was systematically investigated in two identity management modes: verification and identification. The results showed that for a single fixed gesture, using only 0.8-second data, the averaged equal error rate (EER) for verification was 3.5%, and the averaged rank-1 for identification was 90.3%, both comparable to the reported performance of ECG and EEG. The function of customizing gesture code could further improve the verification performance to 1.1% EER. This work demonstrated the potential and effectiveness of sEMG as a biometric trait in user verification and identification, beneficial for the design of future biometric systems.

## Introduction

Biometric systems are used to determine or verify an individual’s identity by measuring his/her physiological and behavioral characteristics that belong uniquely to an individual, i.e. biometric traits. As biometric traits are unique and inherent to each individual, they are difficult to manipulate, share, or forget. As a result, issues such as multiple enrollments by the same person under different identities, or by different persons under the same identities, which traditional methods based on knowledge and tokens are incapable of detecting, can be largely avoided (Jain et al., 2011). Biometric traits greatly improve the reliability of the system in the recognition and authentication of individuals. As such, biometric technology has been extensively used in a large number of applications (Xiao, 2007), including border control, law enforcement, financial security, consumer electronics access control, etc.

The properties of biometric systems mainly depend on the specific traits they use. Fingerprint, iris or retina, and facial features are the three most common biometric traits (Kaur et al., 2014). Systems based on them have already been embedded in our daily lives, such as mobile phones, laptops, and smart pads. However, these traits need to be exposed during recognition, providing the chance to be captured and then synthetically generated. Furthermore, due to poor liveness detection, the improvement in photographic and 3D model reproductions makes the spoofing attacks stronger to circumvent their protections (Pinto et al., 2018). The issues pose serious challenges against system safety and thus, it is necessary to explore the power of other biometric traits.

Recently, there has been a growing interest in the use of electrical biosignals as biometric traits, such as the electrocardiogram (ECG) and electroencephalogram (EEG), the manifestation of electrical activities related to the heart and brain, respectively. Compared to the three common traits mentioned above, the hidden nature of electrical biosignals makes them harder to capture, synthesize, and imitate, and the inherent liveness nature ensures their robustness in distinguishing the artifacts from the real biological targets. There are a number of studies investigating the use of ECG and EEG signals as biometric traits (Campisi and Rocca, 2014; Pinto et al., 2018; Gui et al., 2019). Other than ECG and EEG, the surface electromyogram (sEMG) signal is another typical electrical biosignal, representing the electrical activities of the muscles. However, compared to extensive studies on EEG and ECG, little attention was paid on the application of sEMG in biometrics.

Surface electromyogram signals are normally used as a source signal in hand and wrist gesture recognition (Englehart and Hudgins, 2003). With four to six electrodes attached on the upper limb, the classification accuracy of 10 gestures could reach >95% under the pattern recognition framework (He et al., 2015). However, the high performance is largely limited to the condition that both training and testing data come from the same individual. It is reported that though with the same settings, the control performance would drop significantly when a classifier was trained by the data from one individual and used to predict the gestures from a different individual, implying the existence of individual differences of sEMG features (Matsubara and Morimoto, 2013; Khushaba, 2014). This individual difference brings difficulties in establishing calibration-free sEMG-based gesture recognition. Interestingly, such differences also suggest the possibility of sEMG signals as a potential biometric trait.

In addition to the hidden nature and liveness detection, the high performance of sEMG signals in gesture recognition gives a unique advantage of sEMG biometrics compared to EEG and ECG: the user can customize their codes with different combinations of wrist and hand gestures, like a password. As such, the system can provide two levels of protection, physiology-based and knowledge-based, which is appealing for high-level security targeted applications. In this paper, we systematically investigated the performance of sEMG signal in the person recognition via gesture recognition. This study focused on static gesture and explored the strength of physiology-based and knowledge-based protection, respectively. In the following part, after the elaboration of related work and our contribution, we introduced the data collection of 16 static normal gestures. Then the performance of each gesture in both modes of identity management, verification, and identification, was evaluated. The results of this study demonstrated the feasibility of sEMG signal as a biometric trait and its potential in enhancing the reliability of a biometric system.

### Related Work and Our Contribution

As little attention was received, there was limited literature on sEMG biometrics. In Belgacem et al. (2015), Venugopalan et al. (2015), and Cannan and Hu (2013), sEMG was used as the complementary information and combined with other physiological or behavioral data to strengthen the performance of user authentication. In Yamaba et al. (2018a) and Yamaba et al. (2019), the difference was observed in sEMG amplitudes from the same gesture made by different participants. However, no quantifiable results were concluded. In Shin et al. (2017), Yamaba et al. (2018b), Shioji et al. (2019), and Yamaba et al. (2017), the sEMG signals were used to classify the participants with various types of the classifiers, including artificial neural network (ANN), support vector machine (SVM), convolutional neural network (CNN), and Gaussian mixture model (GMM). However, only a small group of participants (5–11) was investigated, and the study protocol, which focused on participant classification and measured in classification accuracy, were not standard for verification or identification, making the results difficult to be compared with other biometric traits. In summary, it is difficult to infer the power of sEMG biometrics from the results of these studies for weakness in methods.

In this study, we systematically investigated the potential of sEMG as a single biometric trait. For simplicity, only the scenario of the single static gesture was analyzed, and the model training and testing data were from the same day to be consistent with the majority of ECG and EEG studies (Delpozo-Banos et al., 2015; Gutta and Cheng, 2016; Pinto et al., 2018; Wilaiprasitporn et al., 2019). The contribution of this study includes: (1) the performance of sEMG biometrics in both verification and identification was both evaluated and quantified in standard metrics, providing a reference for the future studies; (2) to the best of our knowledge, this was the first study separately evaluating two different powers from sEMG, physiology-based and knowledge-based, for biometrics, which is unique to sEMG-based biometrics.

## Materials and Methods

### Subjects

Twenty-four able-bodied participants (13 males and 11 females, aged from 19 to 30 years old) were recruited in this study. The forearm length (from the wrist to the elbow) was 25.4 ± 1.46 cm. The circumference of the place where the electrodes were attached was 24.5 ± 2.53 cm. Before the experiment, all the subjects read and signed informed consent. The experiment protocol was in accordance with the Declaration of Helsinki and approved by the Office of Research Ethics of the University of Waterloo (ORE#: 22391).

### Data Collection

**FIGURE 1:**
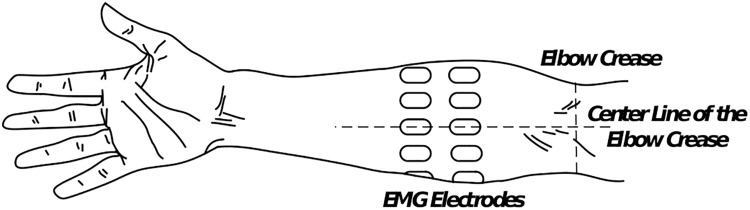
Positions of the 16 monopolar electrodes on the forearm (dorsal view). In each ring, eight electrodes are evenly placed, and one electrode is placed in the centerline of the elbow crease. The distance between the centers of the two rings was 2 cm, and the proximal ring was placed one-third of the forearm length between the elbow crease and the upper ring.

During the experiment, the subject seated comfortably in a height-adjustable chair, naturally extending their arms toward the ground. Sixteen monopolar sEMG electrodes (AM-N00S/E, Ambu, Denmark) were placed in pairs with equal distance around the right forearm, forming two rings as displayed in Figure 1. To decrease the effect of the electrode position variability among the subjects, one of the electrodes in each ring was positioned in the centerline of the elbow crease. The center-to-center distance was 2 cm between the two rings, and the distance from the elbow crease to the center of the upper ring was one-third of the forearm length. Instructions were displayed on the front computer screen to help the participant perform the defined gestures. The sEMG signals were amplified and digitized by a commercial system (EMG USB2+, OT Bioelettronica, Italy). The band-pass filter of the machine was set between 10 and 500 Hz, and the sampling frequency was 1024 Hz. Eight bipolar channels were derived from the data differential of each paired electrodes and obtained by a custom-made software based on Matlab (MathWorks, Inc., United States) platform.

**FIGURE 2:**
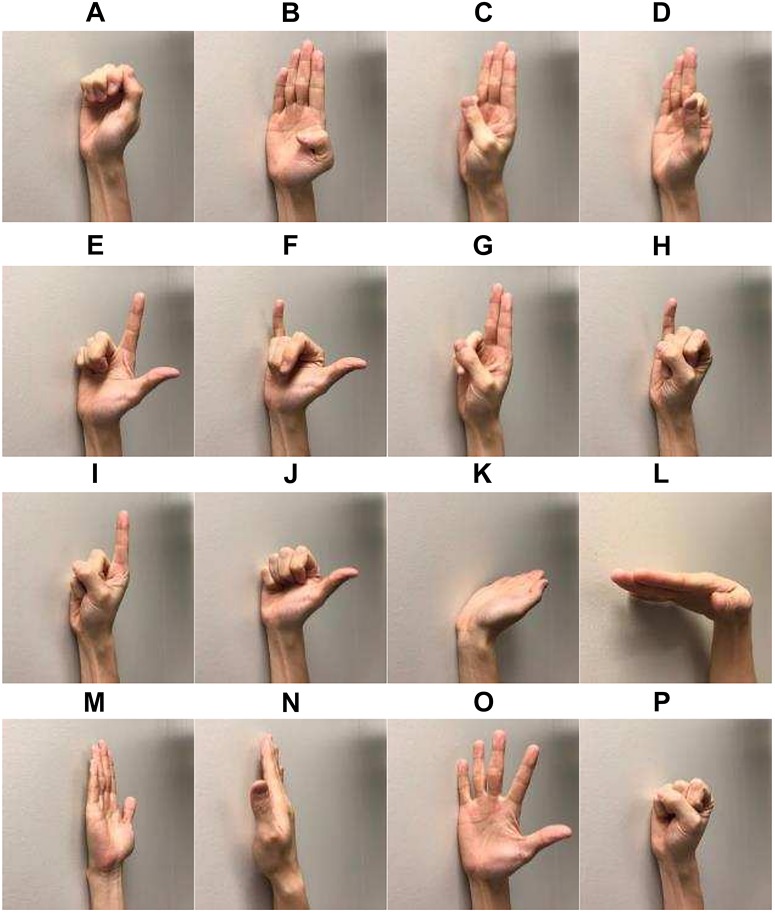
Sixteen gesture classes investigated in the study: **(A)** lateral prehension (LP), **(B)** thumb adduction (TA), **(C)** thumb and little finger opposition (TLFO), **(D)** thumb and index finger opposition (TIFO), **(E)** thumb and little finger extension (TLFE), **(F)** thumb and index finger extension (TIFE), **(G)** index and middle finger extension (IMFE), **(H)** little finger extension (LFE), **(I)** index finger extension (IFE), **(J)** thumb extension (TE), **(K)** wrist flexion (WF), **(L)** wrist extension (WE), **(M)** forearm supination (FS), **(N)** forearm pronation (FP), **(O)** hand open (HO), and **(P)** hand close (HC).

The following 16 hand and wrist gestures were included in the current study (Figure 2): lateral prehension (LP), thumb adduction (TA), thumb and little finger opposition (TLFO), thumb and index finger opposition (TIFO), thumb and little finger extension (TLFE), thumb and index finger extension (TIFE), index and middle finger extension (IMFE), little finger extension (LFE), index finger extension (IFE), thumb extension (TE), wrist flexion (WF), wrist extension (WE), forearm supination (FS), forearm pronation (FP), hand open (HO), and hand close (HC). During the experiment, each gesture was repeated seven times, and the muscle contraction of each repetition was kept for 5 s. To avoid the transient portion of sEMG signals, the subjects were asked to perform each contraction a little bit earlier than the beginning of the recording. There was a 5-s rest period between two consecutive contractions to avoid muscle fatigue. The sequence of the gestures was randomized.

### Evaluation of User Verification

There are two modes of identity management functionalities in biometrics: Verification and identification (Jain et al., 2011). In the Verification mode, the user needs to present his/her biometric data, as well as the identity, to the system. The system compares the input with the template of the claimed identity in the database to verify the authenticity of the claim. In the identification mode, the user only needs to present his/her biometric data. The system compares the input with all the templates in the database and returns one or a couple of identities of which the template has the highest similarity with the input, or a decision stating the user presenting the input is not enrolled. As such, the verification mode is a binary classification, while the identification mode is a multiclass classification. In this study, we investigated both verification and identification modes of the proposed gesture recognition-based biometric system.

To evaluate the verification performance, the raw signal was first windowed, and a robust feature set in myoelectric control, improved Discrete Fourier Transform (iDFT) (Jiayuan et al., 2013), was extracted from each window. It is given by

(1) $${\text{DFT}}_{i}=F\,\left[\frac{1}{N_{i}}\,\sum_{j=1}^{N_{i}}\left|X\,\left(f_{i,j}\right)\right|\right],i=1,2,\ldots ,6$$

where the whole frequency band is equally divided into six segments, and *f*_*i,j*_ denotes the *j*th frequency within the segment *i*. *N*_*i*_ is the total number of points in segment *i*. *X*(*f*) represents the discrete Fourier transform at the frequency *f*, and *F*[⋅] represents logarithmic transformation. The transformation from all the segments and all the channels are concatenated as the feature vector.

The window length was 200 ms. The overlap between two consecutive windows was 150 ms (every 50 ms produces one decision). For a given feature vector sample *p* (the input), its matching score (similarity measurement) with a specific motion class *i* from subject *j* (the template) was defined as the Mahalanobis distance between the sample and the class centroid

(2) $${\text{Score}}_{i,j}\,\left(p\right)=\frac{1}{2}\,\sqrt{{\left(p-\mu_{i,j}\right)}^{\text{T}}\,\sum_{i,j}^{-1}\left(p-\mu_{i,j}\right)}$$

where μ*_*i*_*,*_*j*_* is the centroid of the motion class *i* from subject *j*, and Σ*_*i*_*,*_*j*_* is the covariance matrix. Both parameters are calculated from the system training data, and the sample *p* is from the system testing data.

A decision was made after the comparison between the matching score and the predefined threshold. To increase the verification accuracy, the decision stream was postprocessed by the majority vote. The majority vote decision for a given sample was made from the greatest number of the occurrences in *m* points of the decision stream, which includes *m*−1 previous points. The stream size, *m*, was tested on four levels, 1, 5, 9, and 13. It should be noted that the demand for the delay in biometric was not as critical as that in myoelectric control (<300 ms). As such, a large number of samples for postprocessing was acceptable in the real-world application scenario. Sevenfold cross-validation (CV) was performed. In each iteration of the CV, six-sevenths of the data were used for system training, and the remaining one-seventh was for testing.

The performance of the verification system was evaluated by the detection error tradeoff curve (DEC), where false rejection rate (FRR) was plotted against false acceptance rate (FAR) at various thresholds (for accepting or rejecting the claim). FRR represented the error that the input signal was from the claimed identity, but the claim was rejected. FAR represented the error that the input signal was not from the claimed identity, but the claim was accepted. To provide quantitative assessment of the verification performance: two metrics were derived from DET: equal error rate (EER) and area under curve (AUC). EER refers to the point on the DET curve where FRR equals FAR. AUC refers to the area under the DET curve. The lower the EER and AUC values, the better the performance.

For the sake of simplicity, the code length was limited to one, and each code (gesture) was evaluated independently. Suppose the true user used a specific gesture as the authentication code, for his/her identity, three possible scenarios in the practical applications were simulated to evaluate the verification capability of the proposed system: (1) the authentication code (gesture) was only known to the true user, and other users attempted to claim the identity (the code was not compromised), (2) the code was compromised and other users used the gesture to claim the identity of the true user; (3) and the true user forgot the code, and only the true user attempted to claim the identity by using other gestures. These three scenarios were denoted as *normal test*, *leaked test*, and *self-test*, respectively. In all three scenarios, the genuine data were the code gesture class of the user. On the other hand, in *normal test*, the imposter data were all the gesture classes of all the other users except the true user. In *leaked test*, the imposter data were the known code gesture class of all other users except the true user. In *self-test*, the imposter data were all the gesture classes except the code gesture of the user.

### Evaluation of User Identification

In identification, the signal processing was similar to that in verification. The iDFT feature was extracted after signal windowing. The window length was 200 ms with an overlap of 150 ms. For a given testing sample, the Mahalanobis distance was calculated between the sample and each gesture class of each subject. For a specific distance value, its averaged distance was calculated by averaging the values across *m* points, including its *m*−1 preceding points. The size *m* was tested on four levels, 1, 5, 9, and 13, same in verification.

For performance quantification of the identification mode, rank-*k* identification rate was a common quantity, representing the probability of an identification transaction where the true user’s identity was among the *k* identities returned by the system. It calculates the rate of the correct identity occurring in the top *k* score. In this study, the smaller the averaged distance is, the higher the score is. The value of rank-*k* is an increasing function of *k*. Their relation could be summarized by the cumulative match characteristic (CMC) curve, which plotted rank-*k* against *k*. As the commonly used metrics, the values of Rank-1 and Rank-5 were reported in this study. In addition, sevenfold CV was adopted for each gesture class evaluation. In each iteration, the training and testing data were both from the specific gesture class of all the subjects.

### Statistical Analysis

The purpose of this study was to evaluate the authentication power of each gesture in both verification and identification. As the four measurements, EER, AUC, Rank-1, and Rank-5, all had skewed distributions, the median values, the first and third quartile were reported. The mean value with the optimal parameter was also reported for the comparison with other biometric approaches. The nonparametric Kruskal–Wallis test was adopted to investigate the effect of the postprocessing method (four levels of the sample size) on each measurement. If significance was detected, Tukey’s comparison was performed to assess the difference among the levels. The significance was set as 0.05 in this study.

## Results

### Verification Performance

**FIGURE 3:**
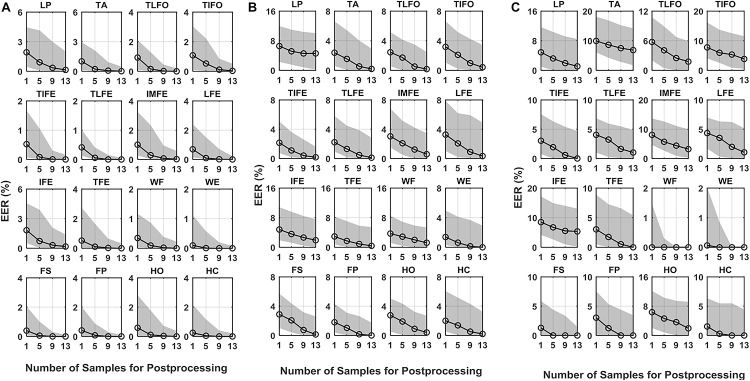
EER of DET for the 16 gestures, LP, TA, TLFO, TIFO, TIFE, TLFE, IMFE, LFE, IFE, TFE, WF, WE, FS, FP, HO, and HC, in three scenarios: **(A)** normal test, **(B)** leaked test, and **(C)** self-test. The dot in each plot represents the median value of EER, and the shaded area represents the range between the first quantile to the third quantile. The scales are different in each plot.

In all the verification scenarios, the EER values decreased with the increase of the number of samples for postprocessing (Figure 3). The rate of decrease with respect to the increasing samples was gradually reduced, as well as the performance difference among the gesture classes. In the *normal test* scenario, the median values of EER without postprocessing (number of samples for postprocessing was equal to 1) were <1.9%. With 13 samples used in postprocessing, the EER range was decreased <0.2%. In the *leaked test* scenario, without postprocessing, the median values of EER ranged from 1.4 to 6.6%. With 13 samples in postprocessing, the EER of LP was 4.6%, and the other EERs were between 1.9 and 0.1%. In *self-test* scenario, without postprocessing, the median values of EER ranged from 9.9 to 3.0%, except for WF (0.6%), WE (0.9%), and FS (1.3%). With 13 samples in postprocessing, the highest EER values were 6.8% (TA), and the EER of eight gestures, TIFE, TLFE, TFE, WF, WE, FS, FP, and HC were <1.0%. For reference, to the best of our knowledge, the performance of ECG and EEG, which was calculated based on the same data settings as this study (training and testing data from the same day), was mainly in the range from 0.1 to 5% (Pinto et al., 2018) and 1 to 20% (Campisi and Rocca, 2014; Fraschini et al., 2015; Gui et al., 2019), respectively, depending on the algorithms and electrode settings (number and positions) adopted.

**FIGURE 4:**
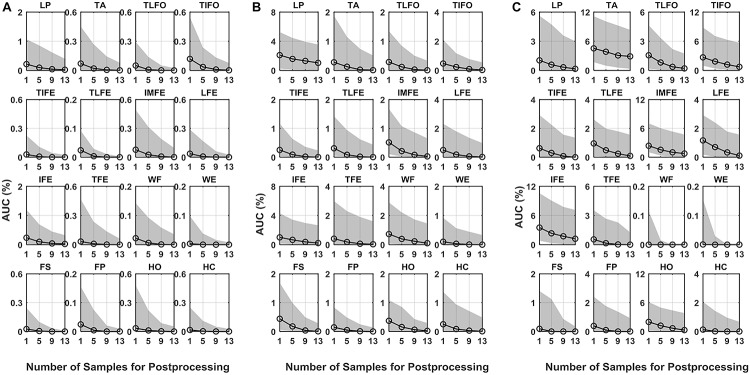
AUC of DET for the 16 gestures, LP, TA, TLFO, TIFO, TIFE, TLFE, IMFE, LFE, IFE, TFE, WF, WE, FS, FP, HO, and HC, in three scenarios: **(A)** normal test, **(B)** leaked test, and **(C)** self-test. The dot in each plot represents the median value of AUC, and the shaded area represents the range between the first quantile to the third quantile. The scales are different in each plot.

**FIGURE 5:**
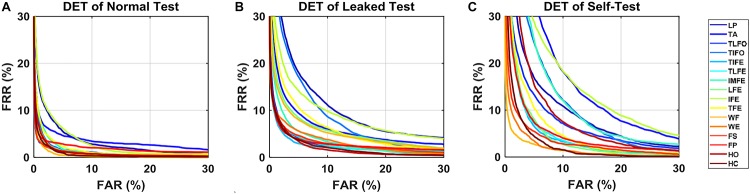
DET curves for each gesture in three scenarios: **(A)** normal test, **(B)** leaking test, and **(C)** self-test. The data are averaged across the subjects.

As for AUC, similar to EER, in all three scenarios, its median values decreased with the increase in the number of postprocessing samples (Figure 4). With 13 postprocessing samples, in *normal test* scenario, the median values of AUC were around 0.0%, while in *leaked test* and *self-test* scenario, the AUC values were <0.2% (except LP: 1.0%), and the <1.2% (except TA: 2.9%), respectively. The average EER and AUC values of each gesture are listed in Table 1. The range of EER for the three scenarios was from 0.6 to 2.2, 2.1 to 7.3, and 1.5 to 9.6%, respectively. Meanwhile, the range of AUC for the three scenarios was from 0.2 to 1.1, 0.7 to 3.8 and 0.6 to 6.1%, respectively. The DET curve of each gesture averaged across all the subjects is presented in Figure 5. The performance in *normal test* was better than the other two, and the performance in *self-test* was the worst.

**TABLE 1:** Verification measurement with 13 samples in postprocessing.

Gesture	Normal test		Leaked test		Self-test	
	EER	AUC	EER	AUC	EER	AUC
LP	**2.2**	**1.1**	**7.3**	3.7	6.0	3.6
TA	1.7	**1.1**	4.0	1.8	**9.6**	**6.1**
TLFO	1.1	0.6	2.4	1.0	5.1	2.6
TIFO	1.0	0.4	2.9	1.2	7.1	4.0
TIFE	0.7	0.3	2.3	1.0	3.6	1.8
TLFE	0.9	0.4	2.6	1.0	4.0	2.0
IMFE	1.1	0.4	2.7	0.9	7.0	4.0
LFE	1.0	0.5	3.6	1.6	3.6	1.8
IFE	2.1	**1.1**	6.9	**3.8**	8.6	5.5
TFE	1.5	0.9	5.3	2.8	3.9	2.1
WF	0.6	0.2	3.7	1.4	1.5	0.6
WE	0.8	0.3	2.7	1.0	1.9	1.0
FS	0.7	0.2	2.4	0.8	3.0	1.3
FP	1.4	0.9	2.8	1.6	3.5	2.0
HO	0.7	0.3	2.1	0.7	5.1	2.5
HC	0.7	0.2	2.2	0.7	2.7	1.2
Average	1.1	0.6	3.5	1.6	4.8	2.6

*All the values are in percentage. The highest value of each test (the worst) are in bold.*

The statistical analysis revealed that the number of samples for postprocessing had a significant effect on the verification performance (EER and AUC) for all the 16 gesture classes (*p* < 0.05). *Post hoc* comparisons with a Bonferroni correction indicated that the values of both EER and AUC, with one sample in postprocessing, was significantly higher than those with 9 and 13 samples in postprocessing; while there was no significant difference between the cases of 9 and 13 samples. For the case of five samples, the difference was not significant compared to the other three cases in both EER and AUC.

### Identification Performance

**FIGURE 6:**
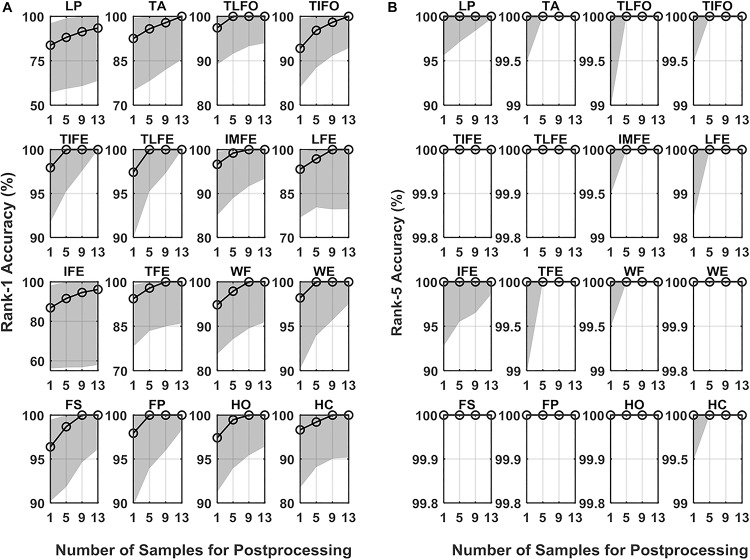
Identification performance measured in **(A)** Rank-1 and **(B)** Rank-5 for the 16 gestures, LP, TA, TLFO, TIFO, TIFE, TLFE, IMFE, LFE, IFE, TFE, WF, WE, FS, FP, HO, and HC, in three scenarios. The dot in each plot represents the median value of AUC, and the shaded area represents the range between the first quantile to the third quantile. The scales are different in each plot.

**FIGURE 7:**
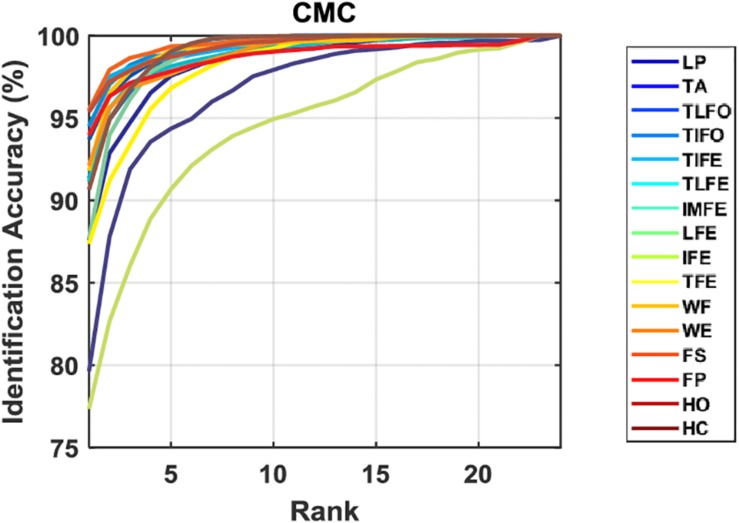
CMC curves for each gesture. The data are averaged across the subjects.

The values of Rank-1 accuracy increased with the increase of the number of samples for postprocessing, but the rate of such increase gradually reduced (Figure 6). Without postprocessing, the median values of Rank-1 ranged from 83.8 to 98.2%. With 13 samples used in postprocessing, the values were close to 100.0% except for LP (93.4%) and IFE (96.2%). In terms of Rank-5 accuracy, the median values of all the gesture classes were close to 100.0% before postprocessing, and the values were kept after postprocessing. For the two gestures with the worst identification performance, LP and IFE, the third quantile values were reduced with the increase of the number of samples for postprocessing. The average Rank-1 and Rank-5 accuracies of each gesture are listed in Table 2. The range of Rank-1 was from 77.3 to 95.5%, and the range of Rank-5 was from 93.4 to 100%. For reference, the Rank-1 performance of ECG and EEG was mainly around 100–80% (Pinto et al., 2018) and 99–80% (Campisi and Rocca, 2014; Fraschini et al., 2015; Gui et al., 2019), respectively. Compared to the other methods, the effect of low identification performance of the two gestures, LP and IFE, could be remedied with the increase of rank number, where the false positive rate increase was retrained for the advantage of multiple codes available in the proposed biometric method. The CMC curve for each gesture is displayed in Figure 7.

**TABLE 2:** Identification measurement with 13 samples in postprocessing.

Gesture	Rank-1	Rank-5
LP	79.6	94.4
TA	87.6	97.6
TLFO	93.7	98.7
TIFO	91.3	98.8
TIFE	94.5	98.9
TLFE	95.5	98.6
IMFE	91.1	98.1
LFE	87.6	98.4
IFE	**77.3**	**90.7**
TFE	87.4	96.8
WF	91.8	99.1
WE	92.1	97.7
FS	95.5	99.4
FP	93.9	97.8
HO	95.4	98.7
HC	90.6	99.0
Average	90.3	97.7

*All the values are in percentage. The highest value of each test (the worst) are in bold.*

Statistical analysis confirmed the effectiveness of the postprocessing on Rank-1 accuracy (*p* < 0.05). For Rank-5 accuracy, the effect of postprocessing was significant on all the gestures except IFE, WE, and FP. The *post hoc* comparison with a Bonferroni correction indicated that there was a significant difference between the values without postprocessing and the values with 13 samples in postprocessing. In addition, the difference between the cases of 9 samples and 13 samples was not significant.

## Discussion

### Performance Comparison With EEG and ECG

This paper systematically investigated the performance of sEMG as a biometric trait in both identity management functions: user identification and user verification. Specifically, in user verification, three scenarios were investigated based on the leakage of the information. Majority vote was adopted and significantly improved the performance of all the cases. Currently, EEG and ECG are two types of electrical biosignals that have been extensively investigated as biometric traits. As alternative electrical biosignals, sEMG-based biometrics has the same two important properties as ECG and EEG: hidden nature and liveness detection, making it more robust to spoofing attacks than traditional biometric traits such as fingerprint and iris. Different from ECG and EEG, sEMG-based biometrics has a unique advantage due to its high accuracy in gesture recognition: enabling users to set different gestures as their own password. Theoretically, EEG-based biometrics could use different mental states to achieve a similar function. However, its number of classes is limited by low classification accuracy. Normally, only two classes could be classified with the accuracy >70% based on EEG (Yao et al., 2014), making it impossible to encode password for real-world applications. The unique property of sEMG provides an additional layer of protection, embedding the knowledge-based method into the biometric system. Such an advantage can be clearly seen in the comparison between *normal test* and *leaked test* of verification. In *normal test*, there are two layers of protection: gesture code and individual difference of sEMG signals, while there is only one layer of protection, individual difference of sEMG signals, in *leaked test*. With the protection from gesture code, the averaged EER and AUC were increased by 2.4 and 1.0%, respectively. For the gesture with the lowest performance, LP, the weakest part of the system, its EER and AUC were increased by 4.9 and 2.6%, respectively. Its performance in *normal test* (EER: 2.2%; AUC: 1.1%) was comparable to the best performance in *leaked test* (EER: 2.1%; AUC: 0.7%). Note that in this current study, we only presented the case of the shortest code length: one gesture. With more gestures, i.e. longer code length, the security level of the system will be improved further.

In *leaked test* of verification and identification, the performance of the proposed system solely depends on the individual difference of sEMG features, which is conceptually similar to EEG- and ECG-based biometric system. In the case of *leaked test* for verification, the averaged EER of sEMG-based biometrics is 3.5%. As mentioned, for the same experimental protocol of this study, which used the training and testing data from the same day, the reported EER of ECG- and EEG-based biometrics was mainly around 0.1–5% (Pinto et al., 2018) and 1–20% (Campisi and Rocca, 2014; Fraschini et al., 2015; Gui et al., 2019), respectively. In terms of identification, the averaged Rank-1 value of sEMG-based biometrics is 90.3%. The reported value of ECG and EEG was mainly in the range from 100 to 80% (Pinto et al., 2018), and 99 to 80% (Campisi and Rocca, 2014; Fraschini et al., 2015; Gui et al., 2019), respectively. It is difficult to provide a precise value here for the difference in a number of factors, such as the electrode placement, the denoising algorithms, features, and classifiers adopted. As such, in the leaked test, the performance of sEMG is comparable to, if not better than, that of EEG and ECG.

Other than the recognition performance, the property of sEMG trait also brings advantages to other aspects. Regarding the data storage, due to the gesture code, the pressure on data security of sEMG-based biometrics is much less than that of EEG- and ECG-based biometrics. For EEG and ECG, there is no option to change the biometric traits when the enrolled identity data are compromised, while the gesture code can be changed if sEMG-based biometrics is used. As such, sEMG-based biometrics requires less on data security, reducing the difficulty and cost of implementation in practical applications. Regarding the response time, though 13 samples were used in majority vote to improve the recognition performance of sEMG trait, for one gesture, the time to generate one decision was only 0.8 s, while ECG needed approximately 1 s (one complete beat), and 5 s recording was recommended for EEG (Delpozo-Banos et al., 2015). The high recognition rate and short response time made sEMG-based biometrics a potential option for the scenarios demanding high-level security and fast reactions, such as banking and financial services, entry, etc.

### Limitation and Future Work

The long-term performance is essential for deploying the biometric system in real life. However, as the inherent non-stationary property, the biometric performance of electrical biosignal traits may be reduced over time. Only a few works of EEG and ECG traits focused on the effect of signal variability using training and testing data from different days. Performance degradation was observed for both ECG- and EEG-based biometrics when the interval between training and testing data was over days (Marcel and del Millan, 2007; Labati et al., 2013). Regarding sEMG, it is expected to have a performance drop in long-term use, unless the system is adaptive. The long-term robustness is an open question for electrical biosignal traits, as well as some behavior traits, such as gait (Matovski et al., 2010; Pataky et al., 2012). Currently, the solution mainly focused on adaptive algorithms to update the model or template (Agrafioti et al., 2012; Pinto et al., 2018). Specifically for sEMG, it has been shown that the signal variability would be decreased with the process of user adaptation (He et al., 2015), which addressed the long-term issue in another direction. It is expected that the performance drop will significantly be reduced in experienced users compared to naive users. The fusion of user adaptation and algorithm adaption will benefit the long-term performance of sEMG-based biometrics.

There are other factors affecting the quality and stability of sEMG signals, such as skin conductance, electrode positions, limb positions, etc. These disturbances will degrade both the performance of verification and identification. In myoelectric control, many methods have been proposed to improve the robustness against these factors (He and Zhu, 2016; Gu et al., 2018), which might be helpful to improve the performance of biometrics. In addition, compared to myoelectric control, identification and verification are short periods, which are feasible to be conducted in a controlled settings to remove or mitigate the effect of these factors.

The advantage of sEMG over ECG and EEG was a repository of gesture codes sEMG provided. These gesture codes enhanced the biometric security of sEMG trait. On the one hand, as mentioned above, the user could customize their own combination of gesture codes to add knowledge-based protection for verification. On the other hand, signal individual difference could be improved by increasing the length of gesture codes. Different gestures may distinguish different groups of users. Suppose in one group, the users have similar signals from one gesture, but different from another. The scenario is opposite for the users of another group. As such, the users from these two groups could be better distinguished with the combination of the two gestures. In addition, the increase of gesture codes may result in a decreased number of qualified users, which is the intersection of the candidate identities of each gesture. To avoid this, the number of candidate identities needs to be increased. With the optimization of the length of gesture code, the performance of sEMG-based biometrics would be improved.

Eight bipolar electrodes were adopted in this study. With the increase of the number of electrodes, more precise information would be collected and the individual difference would be enhanced. In addition, this study used handcrafted features. Deep neural network has been favored for its automatic feature learning ability (He et al., 2019). As its success on myoelectric control (Atzori et al., 2016; Yu et al., 2020), the information of individual difference could be extracted and learned by deep neural networks and the performance of sEMG-based biometrics might be improved.

## Conclusion

This study investigated in detail the performance of sEMG as a biometric trait for both verification and identification. Compared to two traditional electrical biosignal traits, EEG and ECG, sEMG biometrics can provide two layers of identity recognition, one depending on individual signal difference, and another depending on custom-set gesture code. The results indicated that the custom-set gesture code could greatly improve verification performance. If only relying on the inherent signal difference among individuals, the verification and identification performance of sEMG was comparable to that of EEG and ECG reported in the literature. The results of this study would deepen the understanding of sEMG biometrics, and benefit for the design of future biometric systems.

## Data Availability Statement

The raw data supporting the conclusions of this manuscript will be made available by the authors, without undue reservation, to any qualified researcher.

## Ethics Statement

The studies involving human participants were reviewed and approved by the Office of Research Ethics of the University of Waterloo (ORE#: 22391). The patients/participants provided their written informed consent to participate in this study.

## Author Contributions

JH and NJ conceived the study, designed the experiments, performed the data analysis, drafted and revised the manuscript. JH conducted the experiments.

## Conflict of Interest

The authors declare that the research was conducted in the absence of any commercial or financial relationships that could be construed as a potential conflict of interest.

## References

[B1] Agrafioti F. Bui F. M. Hatzinakos D. (2012). secure telemedicine: biometrics for remote and continuous patient verification. *J Comput. Netw. Commun.* 2012 1–11. 10.1155/2012/924791

[B2] Atzori M. Cognolato M. Müller H. (2016). Deep learning with convolutional neural networks applied to electromyography data: a resource for the classification of movements for prosthetic hands. *Front Neurorob.* 10:9. 10.3389/fnbot.2016.00009 27656140 PMC5013051

[B3] Belgacem N. Fournier R. Nait-Ali A. Bereksi-Reguig F. (2015). A novel biometric authentication approach using ECG and EMG signals. *J. Med. Eng. Technol.* 39 226–238. 10.3109/03091902.2015.1021429 25836061

[B4] Campisi P. Rocca D. L. (2014). Brain waves for automatic biometric-based user recognition. *IEEE Trans. Inform. Forensics Secur.* 9 782–800. 10.1109/tifs.2014.2308640

[B5] Cannan J. Hu H. (2013). “Automatic user identification by using forearm biometrics,” in *2013 IEEE/ASME International Conference on Advanced Intelligent Mechatronics: Mechatronics for Human Wellbeing*, (Cranberry Township, PA), 710–715.

[B6] Delpozo-Banos M. Travieso C. M. Weidemann C. T. Alonso J. B. (2015). EEG biometric identification: a thorough exploration of the time-frequency domain. *J. Neural Eng.* 12:056019. 10.1088/1741-2560/12/5/056019 26394698

[B7] Englehart K. Hudgins B. (2003). A robust, real-time control scheme for multifunction myoelectric control. *IEEE Trans. Bio Med. Eng.* 50 848–854. 10.1109/tbme.2003.813539 12848352

[B8] Fraschini M. Hillebrand A. Demuru M. Didaci L. Luca Marcialis G. (2015). An EEG-based biometric system using eigenvector centrality in resting state brain networks. *IEEE Signal Process. Lett.* 22 666–670. 10.1109/lsp.2014.2367091

[B9] Gu Y. Yang D. Huang Q. Yang W. Liu H. (2018). Robust emg pattern recognition in the presence of confounding factors: features, classifiers and adaptive learning. *Expert Syst. Appl.* 96 208–217. 10.1016/j.eswa.2017.11.049

[B10] Gui Q. Ruiz-Blondet M. V. Laszlo S. Jin Z. (2019). A survey on brain biometrics. *ACM Comput. Surv.* 51 1–38.

[B11] Gutta S. Cheng Q. (2016). Joint feature extraction and classifier design for ecg-based biometric recognition. *IEEE J. Biomed. Health Inform* 20 460–468. 10.1109/JBHI.2015.2402199 25680220

[B12] He J. Li K. Liao X. Zhang P. Jiang N. (2019). Real-time detection of acute cognitive stress using a convolutional neural network from electrocardiographic signal. *IEEE Access* 7 42710–42717. 10.1109/access.2019.2907076

[B13] He J. Zhang D. Jiang N. Sheng X. Farina D. Zhu X. (2015). User adaptation in long-term, open-loop myoelectric training: implications for EMG pattern recognition in prosthesis control. *J. Neural Eng.* 12:046005. 10.1088/1741-2560/12/4/046005 26028132

[B14] He J. Zhu X. (2016). Combining improved gray-level co-occurrence matrix with high density grid for myoelectric control robustness to electrode shift. *IEEE Trans. Neural Syst. Rehabil. Eng.* 11 1–1. 10.1109/TNSRE.2016.2644264 28026779

[B15] Jain A. K. Ross A. A. Nandakumar K. (2011). *Introduction to Biometrics.* Berlin: Springer Science & Business Media.

[B16] Jiayuan H. Zhang D. Sheng X. Meng J. Zhu X. (2013). “Improved discrete fourier transform based spectral feature for surface electromyogram signal classification,” in *2013 35th Annual International Conference of the IEEE Engineering in Medicine and Biology Society (EMBC)*, Vol. 2013 (Piscataway, NJ: IEEE), 6897–6900. 10.1109/EMBC.2013.6611143 24111330

[B17] Kaur G. Singh G. Kumar V. (2014). A review on biometric recognition. *Int. J. BioSci. Bio Technol.* 6 69–76.

[B18] Khushaba R. N. (2014). Correlation analysis of electromyogram signals for multiuser myoelectric interfaces. *IEEE Trans. Neural Syst. Rehabil. Eng.* 22 745–755. 10.1109/TNSRE.2014.2304470 24760933

[B19] Labati R. D. Sassi R. Scotti F. (2013). “ECG biometric recognition: permanence analysis of QRS signals for 24 hours continuous authentication,” in *Proceedings of the 2013 IEEE International Workshop on Information Forensics and Security, WIFS*, Vol. 2013 (Piscataway, NJ: IEEE), 31–36.

[B20] Marcel S. del Millan J. R. (2007). Person authentication using brainwaves (EEG) and maximum a posteriori model adaptation. *IEEE Trans. Pattern Anal Mach. Intell.* 29 743–748. 17299229 10.1109/TPAMI.2007.1012

[B21] Matovski D. S. Nixon M. S. Mahmoodi S. Carter J. N. (2010). “The effect of time on the performance of gait biometrics,” in *IEEE 4th International Conference on Biometrics: Theory, Applications and Systems, BTAS*, (Piscataway, NJ: IEEE), 2010.

[B22] Matsubara T. Morimoto J. (2013). Bilinear modeling of EMG signals to extract user-independent features for multiuser myoelectric interface. *IEEE Trans. n Bio Med. Eng.* 60 2205–2213. 10.1109/TBME.2013.2250502 23475334

[B23] Pataky T. C. Mu T. Bosch K. Rosenbaum D. Goulermas J. Y. (2012). Gait recognition: highly unique dynamic plantar pressure patterns among 104 individuals. *J. R. Soc. Interface* 9 790–800. 10.1098/rsif.2011.0430 21900318 PMC3284135

[B24] Pinto J. R. Cardoso J. S. Lourenco A. (2018). Evolution, Current Challenges, And Future Possibilities in ECG biometrics. *IEEE Access* 6 34746–34776. 10.1109/access.2018.2849870

[B25] Shin S. Jung J. Kim Y. T. (2017). 2017 1–3.

[B26] Shioji R. Ito S. I. Ito M. Fukumi M. (2019). “Personal authentication and hand motion recognition based on wrist EMG analysis by a convolutional neural network,” in *Proceedings - 2018 IEEE International Conference on Internet of Things and Intelligence System, IOTAIS*, (Piscataway, NJ: IEEE), 184–188.

[B27] Venugopalan S. Juefei-Xu F. Cowley B. Savvides M. (2015). “Electromyograph and keystroke dynamics for spoof-resistant biometric authentication,” in *IEEE Computer Society Conference on Computer Vision and Pattern Recognition Workshops*, Vol. 2015 (Piscataway, NJ: IEEE), 109–118.

[B28] Wilaiprasitporn T. Ditthapron A. Matchaparn K. Tongbuasirilai T. Banluesombatkul N. Chuangsuwanich E. (2019). “Affective EEG-based person identification using the deep learning approach,” in *IEEE Transactions on Cognitive and Developmental Systems*, (Piscataway, NJ: IEEE).

[B29] Xiao Q. (2007). “Technology review - biometrics-technology, application, challenge, and computational intelligence solutions,” in *IEEE Computational Intelligence Magazine*, Vol. 2 (Piscataway, NJ: IEEE).

[B30] Yamaba H. Aburada K. Katayama T. Park M. Okazaki N. (2018a). “Evaluation of user identification methods for realizing an authentication system using S-EMG,” in *Advances in Network-Based Information Systems. NBiS 2018. Lecture Notes on Data Engineering and Communications Technologies*, Vol. 22 eds Barolli L. Kryvinska N. Enokido T. Takizawa M., (Cham: Springer).

[B31] Yamaba H. Inotani S. Usuzaki S. Takatsuka K. Aburada K. Katayama T. (2019). Introduction of fingerspelling for realizing a user authentication method using S-EMG. *Adv. Intell. Syst. Comput.* 927 93–701.

[B32] Yamaba H. Kurogi T. Aburada K. Kubota S. I. Katayama T. Park M. (2018b). On applying support vector machines to a user authentication method using surface electromyogram signals. *Artif. Life Robot.* 23 87–93. 10.1007/s10015-017-0404-z

[B33] Yamaba H. Kurogi A. Kubota S. I. Katayama T. Park M. Okazaki N. (2017). Evaluation of feature values of surface electromyograms for user authentication on mobile devices. *Artif. Life Robot.* 22 108–112. 10.1007/s10015-016-0323-4

[B34] Yao L. Meng J. Zhang D. Sheng X. Zhu X. (2014). Combining motor imagery with selective sensation toward a hybrid-modality BCI. *IEEE Trans. Biomed. Eng.* 61 2304–2312. 10.1109/TBME.2013.2287245 24235291

[B35] Yu Y. Chen C. Sheng X. Zhu X. (2020). Multi-DoF continuous estimation for wrist torques using stacked autoencoder. *Biomed. Signal Process. Control* 57:101733 10.1016/j.bspc.2019.101733

